# Crystal structure, Hirshfeld surface analysis and HOMO–LUMO analysis of (*E*)-4-bromo-*N*′-(4-meth­oxy­benzyl­idene)benzohydrazide

**DOI:** 10.1107/S2056989018013373

**Published:** 2018-09-28

**Authors:** Kasthuri Balasubramani, Ganesan Premkumar, Palaniyappan Sivajeyanthi, Muthaiah Jeevaraj, Bellarmin Edison, Toka Swu

**Affiliations:** a Department of Chemistry, Government Arts College (Autonomous), Thanthonimalai, Karur 639 005, Tamil Nadu, India; b Department of Chemistry, Pondicherry University, R.V. Nagar, Kalapet, Puducherry 605 014, India

**Keywords:** crystal structure, Schiff base, inter­molecular inter­actions, Hirshfeld surface analysis, HOMO–LUMO calculation

## Abstract

The title Schiff base compound, C_15_H_13_BrN_2_O_2_, displays an *E* configuration with respect to the C=N double bond, which forms a dihedral angle of 58.06 (9)° with the benzene ring. In the crystal, the mol­ecules are linked into chains parallel to the *b* axis by N—H⋯O and C—H⋯O hydrogen bonds, giving rise to rings with an *R*_2_^1^(6) graph-set motif. The chains are further linked into a three-dimensional network by C—H⋯π inter­actions. A Hirshfeld surface analysis indicates that the most important contributions to the crystal packing are from C⋯H (33.2%), H⋯H (27.7%), Br⋯H/H⋯Br (14.2%) and O⋯H/H⋯O (13.6%) inter­actions. The title compound has also been characterized by frontier mol­ecular orbital analysis.

## Abstract 2

The title Schiff base compound displays an *E* configuration with respect to the C=N double bond. The benzene rings form a dihedral angle of 58.06 (9)°. In the crystal, the mol­ecules are linked by N—H⋯O and C—H⋯O hydrogen bonds into chains, which are further connected into a three-dimensional network by C—H⋯π inter­actions.

## Chemical context   

Schiff bases are nitro­gen-containing compounds that were first obtained by the condensation reactions of aromatic amines and aldehydes (Schiff *et al.*, 1864 ▸). A wide range of these compounds with the general formula *R*HC=N*R*_1_ (*R* and *R*_1_ can be alkyl, aryl, cyclo­alkyl or heterocyclic groups) have been synthesized. Schiff bases are of great importance in the field of coordination chemistry because they are able to form stable complexes with metal ions (Souza *et al.*, 1985 ▸). The chemical and biological significance of Schiff bases can be attributed to the presence of a lone electron pair in the *sp* ^2^-hybridized orbital of the nitro­gen atom of the azomethine group (Singh *et al.*, 1975 ▸). These compounds are used in the fields of organic synthesis, chemical catalysis and medicine, pharmacy, as well as other new technologies (Tanaka *et al.*, 2010 ▸). Schiff bases are also used as probes in investigating the structure of DNA (Tiwari *et al.*, 2011 ▸), and have gained special attention in pharmacophore research and in the development of several bioactive lead mol­ecules (Muralisankar *et al.*, 2016 ▸). Schiff bases showing photochromic and thermochromic properties have been used in information storage, electronic display systems, optical switching devices and ophthalmic glasses (Amimoto *et al.*, 2005 ▸). Herein the crystal structure of the title compound, (*E*)-4-bromo-*N*′-(4-meth­oxy­benzyl­idene)benzohydrazide is reported.
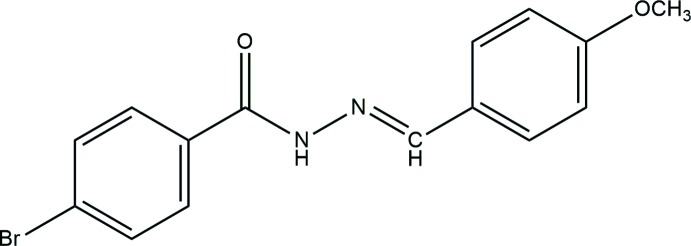


## Structural commentary   

**Figure 1:**
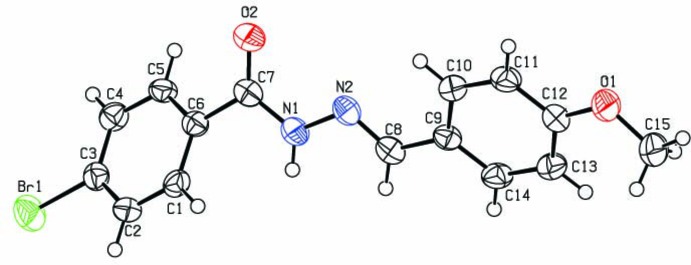
The mol­ecular structure of the title compound with displacement ellipsoids drawn at the 50% probability level.

The asymmetric unit of the title compound (Fig. 1 ▸) consists of one independent mol­ecule displaying an *E* configuration about the C=N double bond. All the bond lengths are within the normal ranges. The values of the C8=N2 [1.281 (3) Å] and C7=O2 [1.222 (3) Å] bond lengths confirm their double-bond character. The C7—N1, N1—N2 and C3—Br1 bond lengths are 1.354 (3), 1.379 (3) and 1.894 (3) Å, respectively. The central O2/C7/N1/N2 fragment is approximately planar (r.m.s. deviation 0.0141 Å) and forms dihedral angles of 32.5 (2) and 27.2 (2)° with the C1–C6 and C9–C14 rings, respectively. The dihedral angle formed by the aromatic rings is 58.06 (9)°.

## Supra­molecular features   

**Figure 2:**
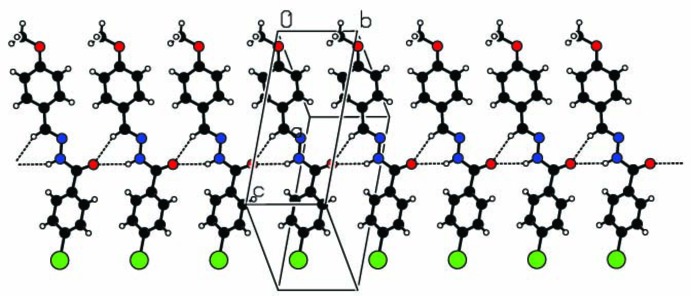
Partial packing diagram of the title compound showing the formation of a mol­ecular chain parallel to the *b* axis through N—H⋯O and C—H⋯O hydrogen bonds (dashed lines).

**Figure 3:**
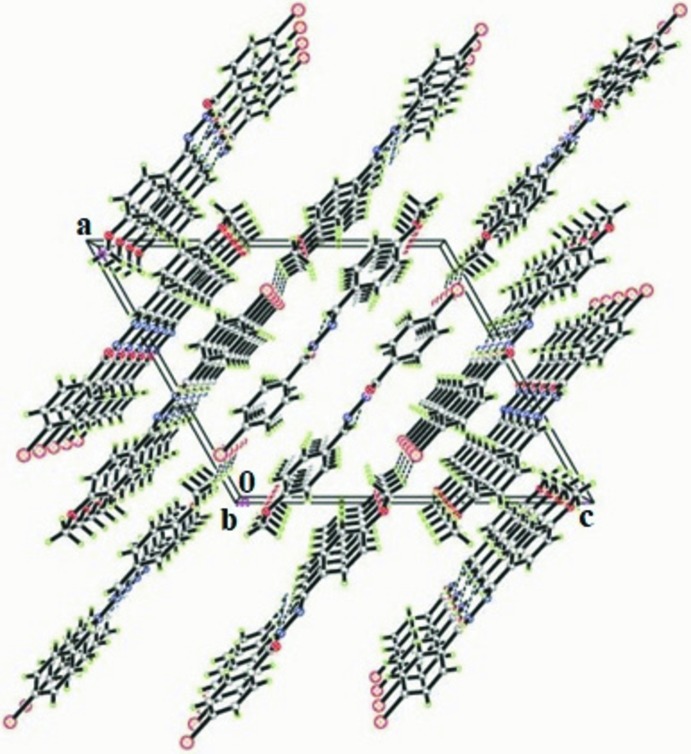
Packing diagram of the title compound viewed down the *b* axis.

In the crystal structure, the mol­ecules are linked into chains extending along the *b*-axis direction by N1—H1*N*⋯O2 and C8—H8⋯O2 hydrogen-bonding inter­actions (Table 1 ▸) forming rings with an 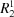
(6) graph-set motif (Fig. 2 ▸). The chains are further connected by C—H⋯π inter­actions, forming a three-dimensional network (Fig. 3 ▸).

## Hirshfeld surface analysis   

**Figure 4:**
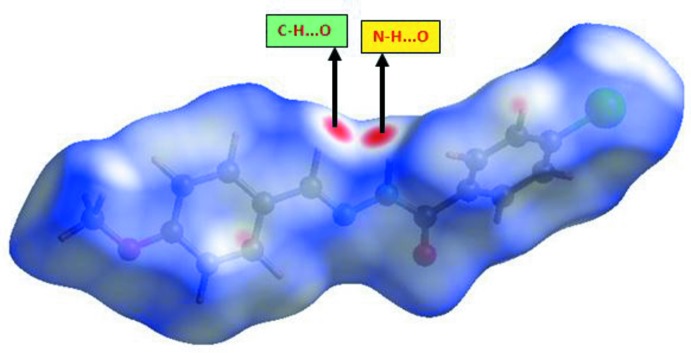
Hirshfeld surfaces of the title compound mapped over *d*_norm_.

**Figure 5:**
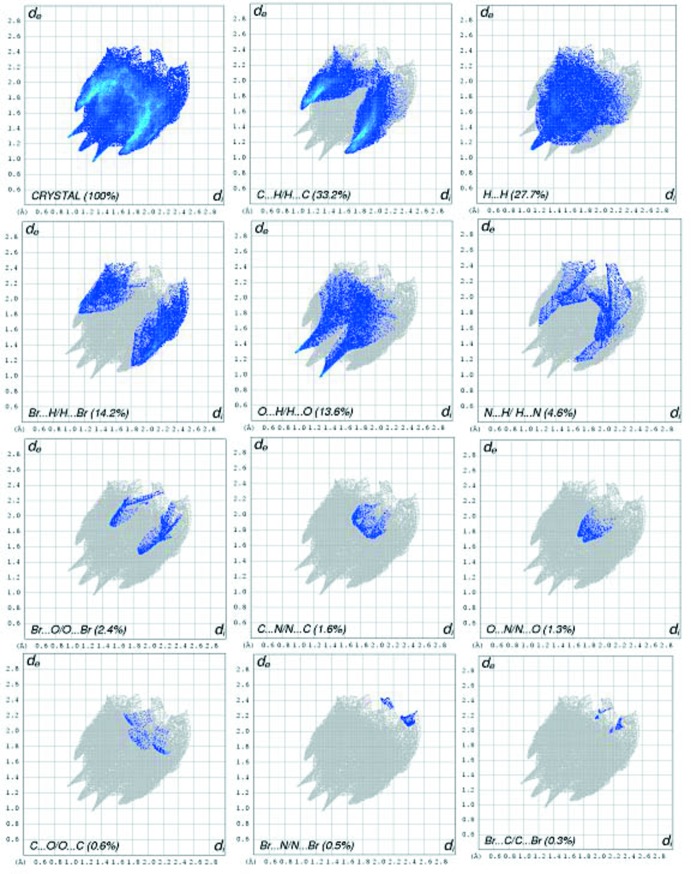
Two-dimensional fingerprint plots of the title compound and relative contributions of the atom pairs to the Hirshfeld surface.

The three-dimensional *d*_norm_ surface is a useful tool for analysing and visualizing the inter­molecular inter­actions. *d*_norm_ takes negative or positive values depending on whether the inter­molecular contact is shorter or longer, respectively, than the van der Waals radii (Spackman & Jayatilaka, 2009 ▸; McKinnon *et al.*, 2007 ▸). The three-dimensional *d*_norm_ surface of the title compound is shown in Fig. 4 ▸. The red points, which represent closer contacts and negative *d*_norm_ values on the surface, correspond to the N—H⋯O and C—H⋯O inter­actions. Two-dimensional fingerprint plots from Hirshfeld surface analysis (Fig. 5 ▸) provide information about the inter­molecular contacts and their percentage contributions to the Hirshfeld surface. The percentage contributions from the different inter­atomic contacts to the Hirshfeld surface in the title compound are as follows: C⋯H (33.2%), H⋯H (27.7%), Br⋯H/H⋯Br (14.2%), O⋯H/H⋯O (13.6%), N⋯H/H⋯N (4.6%), Br⋯O/O⋯Br (2.4%), C⋯N/N⋯C (1.6%), O⋯N/N⋯O (1.3%), O⋯C/C⋯O (0.6%), Br⋯N/N⋯Br (0.5%) and Br⋯C/C⋯Br (0.3%).

## Frontier mol­ecular orbitals   

**Figure 6:**
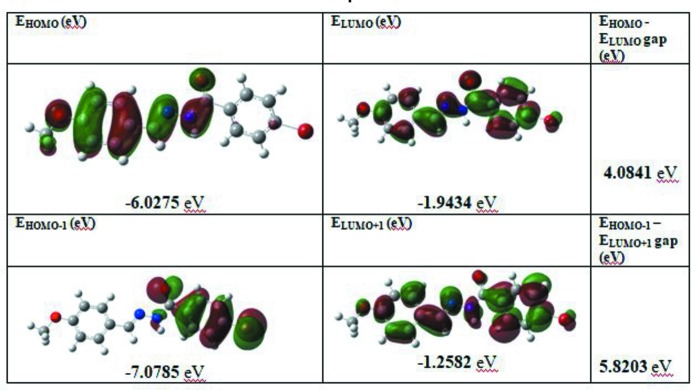
Mol­ecular orbital energy levels of the title compound.

The HOMO (highest occupied mol­ecular orbital) acts as an electron donor and the LUMO (lowest occupied mol­ecular orbital) acts as an electron acceptor. If the energy gap is small then the mol­ecule is highly polarizable and has high chemical reactivity. The energy levels were computed by the DFT-B3LYP/6-311G++(d,p) method (Becke, 1993 ▸) as implemented in *GAUSSIAN09* (Frisch *et al.*, 2009 ▸). The electron distribution of the HOMO-1, HOMO, LUMO and LUMO+1 energy levels, which determines the chemical stability, chemical hardness, chemical potential, electronegativity and electrophilicity index (Table 2 ▸), are shown in Fig. 6 ▸. The frontier mol­ecular orbital LUMO is located over the whole of the mol­ecule. The energy gap of the mol­ecule clearly shows the charge-transfer inter­action involving donor and acceptor groups. From the HOMO–LUMO energy gap, information on whether or not the mol­ecule is difficult (hard) or delicate (soft) can be derived. If the mol­ecule has a large energy gap, then the mol­ecule can be defined as a hard mol­ecule whereas the presence of a small energy gap classifies the mol­ecule as soft. The soft mol­ecules are more polarizable than the hard ones because they only need a small energy for excitation. Therefore, from the data reported in Table 2 ▸, we conclude that the mol­ecule of the title compound belongs to the really hard materials.

## Database survey   

A search of the Cambridge Structural Database (Version 5.39, update May 2018; Groom *et al.*, 2016 ▸) for uncoordinated mol­ecules containing the 4-bromo­benzohydrazide fragment yielded 17 hits. Similar to the crystal structure of the title compound, in seven of them the carbonyl oxygen atom is engaged in inter­molecular N—H⋯O and C—H⋯O hydrogen bonds as a bifurcated acceptor [4-bromo-*N*′-(2,4-di­hydroxy­benzyl­idene)benzohydrazide (Mohanraj *et al.*, 2016 ▸; Arunagiri *et al.*, 2018 ▸); 4-bromo-*N*′-(2-nitro­benzyl­idene)benzohydrazide (Zhang *et al.* 2009 ▸); 4-bromo-*N*′-(2-hy­droxy-5-meth­oxy­benzyl­idene)benzohydrazide (Wang *et al.*, 2017 ▸)] or trifurcated acceptor [4-bromo-*N*′-(2-chloro­benzyl­idene)benzohydrazide (Shu *et al.*, 2009 ▸); 4-bromo-*N*′((5-methyl­furan-2-yl)methyl­ene)benzohydrazide (Bai & Jing, 2007 ▸); 4-bromo-*N*′-(4-methyl-1,2,3-thia­dizole-5-yl)methyl­idenebenzohydrazine (Zhang *et al.*, 2017 ▸); (2-fluoro-2-methyl-2-phenyl­ethyl­idene) 4-bromo­benzoyl hydrazone (Brandes *et al.*, 2006 ▸)], forming mol­ecular chains.

## Synthesis and crystallization   

The title compound was synthesized by the reaction of a 1:1 molar ratio mixture of a hot ethano­lic solution (20 mL) of 4-bromo­benzohydrazide (0.213 mg) and a hot ethano­lic solution of 4-meth­oxy­benzaldehyde (0.136 mg). The mixture was refluxed for 8 h, then it was cooled and kept at room temperature. The powder formed was recrystallized from DMSO. Colourless block-shaped crystals suitable for X-ray analysis were obtained after a few days on slow evaporation of the solvent.

## Refinement   

Crystal data, data collection and structure refinement details are summarized in Table 3 ▸. The hydrogen atoms were positioned geometrically (C—H = 0.93–0.9 Å, N—H = 0.86 Å) and were refined as riding with *U*_iso_(H) = 1.2*U*_eq_(C, N) or 1.5*U*_eq_(C) for methyl H atoms. A rotating model was used for the methyl H atoms. Three outliers (100, 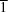
02, 002) were omitted in the last cycles of refinement.

## Supplementary Material

Crystal structure: contains datablock(s) global, I, 1. DOI: 10.1107/S2056989018013373/rz5241sup1.cif

Structure factors: contains datablock(s) I. DOI: 10.1107/S2056989018013373/rz5241Isup2.hkl

Supporting information file. DOI: 10.1107/S2056989018013373/rz5241Isup3.cml

CCDC reference: 1587248

Additional supporting information:  crystallographic information; 3D view; checkCIF report

## Figures and Tables

**Table 1:** Hydrogen-bond geometry (Å, °) *Cg*1 and *Cg*2 are the centroids of the C1–C6 and C9–C14 rings, respectively.

*D*—H⋯*A*	*D*—H	H⋯*A*	*D*⋯*A*	*D*—H⋯*A*
N1—H1*N*⋯O2^i^	0.86	2.40	3.193 (3)	154
C8—H8⋯O2^i^	0.93	2.43	3.240 (3)	146
C2—H2⋯*Cg*2^ii^	0.93	2.81	3.531 (4)	135
C5—H5⋯*Cg*1^iii^	0.93	2.89	3.553 (4)	130
C10—H10⋯*Cg*1^iv^	0.93	2.86	3.549 (4)	132

Symmetry codes: (i) 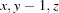; (ii) 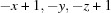; (iii) 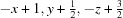; (iv) 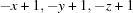.

**Table 2:** Calculated frontier mol­ecular orbital energies (eV)

FMO	Energy
*E*_HOMO_	−6.0275
*E*_LUMO_	−1.9434
*E*_HOMO−1_	−7.0785
*E*_LUMO+1_	−1.2582
*(E*_HOMO_ − *E*_LUMO_) gap	4.0841
*(E*_HOMO−1_ − *E*_LUMO+1_) gap	5.8203
Chemical hardness	2.0420
Chemical potential	3.9854
Electronegativity	−3.9854
Electrophilicity index	3.8892

**Table 3:** Experimental details

Crystal data	
Chemical formula	C_15_H_13_BrN_2_O_2_
*M*_r_	333.18
Crystal system, space group	Monoclinic, *P*2_1_/*c*
Temperature (K)	296
*a*, *b*, *c* (Å)	15.6963 (14), 5.4121 (4), 18.6224 (16)
β (°)	119.609 (6)
*V* (Å^3^)	1375.4 (2)
*Z*	4
Radiation type	Mo *K*α
μ (mm^−1^)	2.99
Crystal size (mm)	0.30 × 0.20 × 0.20

Data collection	
Diffractometer	Bruker Kappa APEXII CCD
Absorption correction	Multi-scan (*SADABS*; Bruker, 2004 ▸)
*T*_min_, *T*_max_	0.467, 0.586
No. of measured, independent and observed [*I* > 2σ(*I*)] reflections	9456, 2563, 1923
*R*_int_	0.030
(sin θ/λ)_max_ (Å^−1^)	0.606

Refinement	
*R*[*F* ^2^ > 2σ(*F* ^2^)], *wR*(*F* ^2^), *S*	0.032, 0.073, 1.02
No. of reflections	2563
No. of parameters	182
H-atom treatment	H-atom parameters constrained
Δρ_max_, Δρ_min_ (e Å^−3^)	0.46, −0.49

Computer programs: *APEX2*, *SAINT* and *XPREP* (Bruker, 2004 ▸), *SIR92* (Altomare *et al.*, 1999 ▸), *SHELXL2017/1* (Sheldrick, 2015 ▸), *ORTEP-3 for Windows* (Farrugia, 2012 ▸) and *Mercury* (Macrae *et al.*, 2008 ▸).

## supplementary crystallographic information

### Crystal data

**Table:**

C_15_H_13_BrN_2_O_2_	*F*(000) = 672
*M**_r_* = 333.18	*D*_x_ = 1.609 Mg m^−^^3^
Monoclinic, *P*2_1_/*c*	Mo *K*α radiation, λ = 0.71073 Å
*a* = 15.6963 (14) Å	Cell parameters from 3040 reflections
*b* = 5.4121 (4) Å	θ = 6.0–48.0°
*c* = 18.6224 (16) Å	µ = 2.99 mm^−^^1^
β = 119.609 (6)°	*T* = 296 K
*V* = 1375.4 (2) Å^3^	Block, colourless
*Z* = 4	0.30 × 0.20 × 0.20 mm

### Data collection

**Table:**

Bruker Kappa APEX2 CCD diffractometer	1923 reflections with *I* > 2σ(*I*)
Radiation source: fine-focus sealed tube	*R*_int_ = 0.030
ω and φ scan	θ_max_ = 25.5°, θ_min_ = 2.8°
Absorption correction: multi-scan (SADABS; Bruker, 2004)	*h* = −19→18
*T*_min_ = 0.467, *T*_max_ = 0.586	*k* = −6→6
9456 measured reflections	*l* = −20→22
2563 independent reflections	

### Refinement

**Table:**

Refinement on *F*^2^	Primary atom site location: structure-invariant direct methods
Least-squares matrix: full	Secondary atom site location: difference Fourier map
*R*[*F*^2^ > 2σ(*F*^2^)] = 0.032	Hydrogen site location: mixed
*wR*(*F*^2^) = 0.073	H-atom parameters constrained
*S* = 1.02	*w* = 1/[σ^2^(*F*_o_^2^) + (0.0247*P*)^2^ + 1.0475*P*] where *P* = (*F*_o_^2^ + 2*F*_c_^2^)/3
2563 reflections	(Δ/σ)_max_ = 0.002
182 parameters	Δρ_max_ = 0.46 e Å^−^^3^
0 restraints	Δρ_min_ = −0.49 e Å^−^^3^

### Special details

**Table:**

Geometry. All esds (except the esd in the dihedral angle between two l.s. planes) are estimated using the full covariance matrix. The cell esds are taken into account individually in the estimation of esds in distances, angles and torsion angles; correlations between esds in cell parameters are only used when they are defined by crystal symmetry. An approximate (isotropic) treatment of cell esds is used for estimating esds involving l.s. planes.

### Fractional atomic coordinates and isotropic or equivalent isotropic displacement parameters (Å^2^)

**Table:**

	*x*	*y*	*z*	*U*_iso_*/*U*_eq_	
Br1	0.79167 (2)	0.30890 (7)	0.92827 (2)	0.05897 (14)	
O1	−0.00864 (14)	0.0494 (4)	0.09196 (11)	0.0533 (5)	
O2	0.43709 (15)	0.7268 (4)	0.55090 (12)	0.0549 (5)	
N1	0.41072 (16)	0.3124 (4)	0.53160 (13)	0.0409 (5)	
H1N	0.427566	0.168774	0.554028	0.049*	
N2	0.33475 (16)	0.3322 (4)	0.45146 (13)	0.0407 (6)	
C1	0.59886 (19)	0.2470 (5)	0.67875 (17)	0.0372 (6)	
H1	0.586962	0.133251	0.637387	0.045*	
C2	0.67360 (19)	0.2047 (5)	0.75843 (16)	0.0381 (6)	
H2	0.712459	0.064141	0.770697	0.046*	
C3	0.68987 (18)	0.3718 (5)	0.81921 (15)	0.0355 (6)	
C4	0.63465 (19)	0.5837 (5)	0.80198 (16)	0.0382 (6)	
H4	0.646969	0.696761	0.843605	0.046*	
C5	0.56079 (19)	0.6261 (5)	0.72216 (16)	0.0365 (6)	
H5	0.523578	0.769580	0.710036	0.044*	
C6	0.54134 (18)	0.4582 (5)	0.65996 (15)	0.0322 (6)	
C7	0.45937 (19)	0.5147 (5)	0.57559 (16)	0.0379 (7)	
C8	0.28735 (19)	0.1307 (5)	0.42189 (16)	0.0390 (7)	
H8	0.304278	−0.008120	0.455644	0.047*	
C9	0.20794 (19)	0.1129 (5)	0.33719 (16)	0.0342 (6)	
C10	0.19274 (19)	0.2921 (5)	0.27816 (16)	0.0379 (6)	
H10	0.232143	0.432276	0.293687	0.046*	
C11	0.1207 (2)	0.2643 (5)	0.19783 (16)	0.0410 (7)	
H11	0.112410	0.384465	0.159190	0.049*	
C12	0.05982 (19)	0.0593 (5)	0.17324 (16)	0.0379 (6)	
C13	0.0727 (2)	−0.1177 (5)	0.23154 (17)	0.0411 (7)	
H13	0.031518	−0.254468	0.216485	0.049*	
C14	0.1471 (2)	−0.0895 (5)	0.31222 (16)	0.0407 (7)	
H14	0.156194	−0.210907	0.350718	0.049*	
C15	−0.0603 (2)	−0.1750 (6)	0.06035 (19)	0.0606 (9)	
H15A	−0.014576	−0.309608	0.077155	0.091*	
H15B	−0.096720	−0.167091	0.001142	0.091*	
H15C	−0.104507	−0.200227	0.081334	0.091*	

### Atomic displacement parameters (Å^2^)

**Table:**

	*U*^11^	*U*^22^	*U*^33^	*U*^12^	*U*^13^	*U*^23^
Br1	0.0530 (2)	0.0698 (2)	0.03805 (19)	0.00375 (18)	0.01023 (14)	0.00735 (16)
O1	0.0519 (12)	0.0497 (13)	0.0362 (11)	−0.0031 (11)	0.0050 (10)	0.0048 (10)
O2	0.0543 (13)	0.0428 (13)	0.0510 (13)	0.0078 (10)	0.0132 (10)	0.0112 (10)
N1	0.0423 (13)	0.0417 (14)	0.0299 (12)	0.0040 (12)	0.0113 (10)	0.0069 (11)
N2	0.0389 (13)	0.0491 (16)	0.0274 (12)	0.0054 (12)	0.0112 (10)	0.0062 (11)
C1	0.0391 (15)	0.0326 (15)	0.0418 (16)	−0.0027 (12)	0.0213 (13)	−0.0107 (12)
C2	0.0346 (14)	0.0325 (15)	0.0426 (16)	0.0040 (13)	0.0156 (13)	0.0017 (13)
C3	0.0316 (14)	0.0403 (16)	0.0324 (15)	−0.0032 (12)	0.0142 (12)	0.0038 (12)
C4	0.0415 (16)	0.0362 (15)	0.0395 (16)	−0.0032 (14)	0.0218 (13)	−0.0060 (13)
C5	0.0377 (15)	0.0280 (14)	0.0460 (16)	0.0063 (12)	0.0224 (13)	0.0035 (12)
C6	0.0319 (14)	0.0319 (14)	0.0356 (15)	0.0010 (12)	0.0187 (12)	0.0034 (12)
C7	0.0363 (15)	0.0397 (18)	0.0395 (16)	0.0043 (14)	0.0202 (13)	0.0045 (13)
C8	0.0400 (16)	0.0409 (17)	0.0359 (15)	0.0065 (14)	0.0185 (13)	0.0065 (13)
C9	0.0361 (15)	0.0344 (15)	0.0336 (14)	0.0059 (12)	0.0184 (12)	0.0019 (12)
C10	0.0394 (15)	0.0317 (14)	0.0429 (16)	−0.0008 (13)	0.0205 (13)	0.0015 (13)
C11	0.0464 (17)	0.0343 (16)	0.0382 (16)	0.0039 (13)	0.0179 (14)	0.0099 (12)
C12	0.0361 (15)	0.0385 (16)	0.0384 (16)	0.0043 (14)	0.0178 (13)	0.0022 (13)
C13	0.0435 (17)	0.0345 (15)	0.0442 (17)	−0.0034 (13)	0.0208 (14)	0.0013 (13)
C14	0.0520 (18)	0.0333 (15)	0.0377 (16)	0.0033 (14)	0.0229 (14)	0.0084 (13)
C15	0.0538 (19)	0.055 (2)	0.0473 (19)	−0.0075 (17)	0.0054 (16)	−0.0005 (16)

### Geometric parameters (Å, º)

**Table:**

Br1—C3	1.894 (3)	C5—H5	0.9300
O1—C12	1.357 (3)	C6—C7	1.490 (4)
O1—C15	1.416 (3)	C8—C9	1.452 (4)
O2—C7	1.222 (3)	C8—H8	0.9300
N1—C7	1.354 (3)	C9—C14	1.375 (4)
N1—N2	1.379 (3)	C9—C10	1.395 (4)
N1—H1N	0.8600	C10—C11	1.367 (4)
N2—C8	1.281 (3)	C10—H10	0.9300
C1—C2	1.382 (4)	C11—C12	1.386 (4)
C1—C6	1.390 (3)	C11—H11	0.9300
C1—H1	0.9300	C12—C13	1.386 (4)
C2—C3	1.371 (4)	C13—C14	1.382 (4)
C2—H2	0.9300	C13—H13	0.9300
C3—C4	1.376 (4)	C14—H14	0.9300
C4—C5	1.380 (4)	C15—H15A	0.9600
C4—H4	0.9300	C15—H15B	0.9600
C5—C6	1.382 (4)	C15—H15C	0.9600
			
C12—O1—C15	118.2 (2)	N2—C8—H8	119.1
C7—N1—N2	121.3 (2)	C9—C8—H8	119.1
C7—N1—H1N	119.4	C14—C9—C10	117.9 (2)
N2—N1—H1N	119.3	C14—C9—C8	120.0 (2)
C8—N2—N1	114.0 (2)	C10—C9—C8	122.0 (2)
C2—C1—C6	120.5 (2)	C11—C10—C9	120.9 (3)
C2—C1—H1	119.7	C11—C10—H10	119.6
C6—C1—H1	119.7	C9—C10—H10	119.6
C3—C2—C1	119.3 (2)	C10—C11—C12	120.8 (2)
C3—C2—H2	120.3	C10—C11—H11	119.6
C1—C2—H2	120.3	C12—C11—H11	119.6
C2—C3—C4	121.2 (2)	O1—C12—C13	125.1 (3)
C2—C3—Br1	118.7 (2)	O1—C12—C11	115.9 (2)
C4—C3—Br1	120.0 (2)	C13—C12—C11	119.0 (2)
C3—C4—C5	119.1 (2)	C14—C13—C12	119.5 (3)
C3—C4—H4	120.4	C14—C13—H13	120.2
C5—C4—H4	120.4	C12—C13—H13	120.2
C4—C5—C6	120.9 (2)	C9—C14—C13	121.9 (2)
C4—C5—H5	119.6	C9—C14—H14	119.1
C6—C5—H5	119.6	C13—C14—H14	119.1
C5—C6—C1	118.9 (2)	O1—C15—H15A	109.5
C5—C6—C7	117.8 (2)	O1—C15—H15B	109.5
C1—C6—C7	123.3 (2)	H15A—C15—H15B	109.5
O2—C7—N1	124.1 (3)	O1—C15—H15C	109.5
O2—C7—C6	121.8 (3)	H15A—C15—H15C	109.5
N1—C7—C6	114.0 (2)	H15B—C15—H15C	109.5
N2—C8—C9	121.9 (2)		

### Hydrogen-bond geometry (Å, º)

Cg1 and Cg2 are the centroids of the C1–C6 and C9–C14 rings, respectively.

**Table:**

*D*—H···*A*	*D*—H	H···*A*	*D*···*A*	*D*—H···*A*
N1—H1*N*···O2^i^	0.86	2.40	3.193 (3)	154
C8—H8···O2^i^	0.93	2.43	3.240 (3)	146
C2—H2···*Cg*2^ii^	0.93	2.81	3.531 (4)	135
C5—H5···*Cg*1^iii^	0.93	2.89	3.553 (4)	130
C10—H10···*Cg*1^iv^	0.93	2.86	3.549 (4)	132

Symmetry codes: (i) *x*, *y*−1, *z*; (ii) −*x*+1, −*y*, −*z*+1; (iii) −*x*+1, *y*+1/2, −*z*+3/2; (iv) −*x*+1, −*y*+1, −*z*+1.
